# An entire exon 3 germ-line rearrangement in the *BRCA2 *gene: pathogenic relevance of exon 3 deletion in breast cancer predisposition

**DOI:** 10.1186/1471-2350-12-121

**Published:** 2011-09-22

**Authors:** Danièle Muller, Etienne Rouleau, Inès Schultz, Sandrine Caputo, Cédrick Lefol, Ivan Bièche, Olivier Caron, Catherine Noguès, Jean Marc Limacher, Liliane Demange, Rosette Lidereau, Jean Pierre Fricker, Joseph Abecassis

**Affiliations:** 1 Division of oncogenetic, Department of Biology and Pathology, Regional Cancer Centre Paul Strauss, BP30042, 67065 Strasbourg, France; 2 Oncogenetic laboratory, Institut Curie-Hospital René Huguenin, 92210 Saint Cloud, France; 3 Department of oncology, Hôpitaux Universitaires de Strasbourg, 67071 Strasbourg, France; 4 Clinical oncogenetic department, Institut Curie-Hospital René Huguenin, 92210 Saint Cloud, France

## Abstract

**Background:**

Germ-line mutations in the *BRCA1 *and *BRCA2 *genes are major contributors to hereditary breast/ovarian cancer. Large rearrangements are less frequent in the *BRCA2 *gene than in *BRCA1*. We report, here, the first total deletion of exon 3 in the *BRCA2 *gene that was detected during screening of 2058 index cases from breast/ovarian cancer families for *BRCA2 *large rearrangements. Deletion of exon 3, which is in phase, does not alter the reading frame. Low levels of alternative transcripts lacking exon 3 (Δ3 delta3 transcript) have been reported in normal tissues, which raises the question whether deletion of exon 3 is pathogenic.

**Methods:**

Large *BRCA2 *rearrangements were analysed by QMPSF (Quantitative Multiplex PCR of Short Fluorescent Fragments) or MLPA (Multiplex Ligation-Dependent Probe Amplification). The exon 3 deletion was characterized with a "zoom-in" dedicated CGH array to the *BRCA2 *gene and sequencing. To determine the effect of exon 3 deletion and assess its pathogenic effect, three methods of transcript quantification were used: fragment analysis of FAM-labelled PCR products, specific allelic expression using an intron 2 polymorphism and competitive quantitative RT-PCR.

**Results:**

Large rearrangements of *BRCA2 *were detected in six index cases out of 2058 tested (3% of all deleterious *BRCA2 *mutations). This study reports the first large rearrangement of the *BRCA2 *gene that includes all of exon 3 and leads to an *in frame *deletion of exon 3 at the transcriptional level. Thirty five variants in exon 3 and junction regions of *BRCA2 *are also reported, that contribute to the interpretation of the pathogenicity of the deletion. The quantitative approaches showed that there are three classes of delta3 *BRCA2 *transcripts (low, moderate and exclusive). Exclusive expression of the delta3 transcript by the mutant allele and segregation data provide evidence for a causal effect of the exon 3 deletion.

**Conclusion:**

This paper highlights that large rearrangements and total deletion of exon 3 in the *BRCA2 *gene could contribute to hereditary breast and/or ovarian cancer. In addition, our findings suggest that, to interpret the pathogenic effect of any variants of exon 3, both accurate transcript quantification and co-segregation analysis are required.

## Background

The *BRCA2 *gene (MIM#600185) is a tumour suppressor gene that codes for a 3,418 amino-acid protein. It is involved in DNA damage repair through homologous recombination, chromatin remodelling and regulation of transcription [1]. All of these functions are important for the maintenance of genome integrity. Germ-line mutations in the *BRCA2 *gene predispose to high risk of breast and ovarian cancer (BOC). Mean cumulative risks for breast and ovarian cancer in *BRCA2 *mutation carriers are 49% and less than 20% at 70 years of age, respectively [2].

Genetic testing is now performed in routine for women with severe family histories of breast and ovarian cancer, in order to identify deleterious mutations in the two susceptibility genes, *BRCA1 *and *BRCA2*. Point mutations account for about 20% of families, depending on the inclusion criteria. Tests for *BRCA1 *gene rearrangements have been added recently, as large rearrangements contribute to ~10% of the *BRCA1 *mutations detected. In contrast to the *BRCA1 *gene, reports of large rearrangements in the *BRCA2 *gene are rare. Up to now, seven large rearrangements of *BRCA2 *have been described in France [3].

In our two French Medical Centres (St Cloud and Strasbourg), a retrospective screening for large rearrangements in the *BRCA2 *gene on 2058 index cases that lacked *BRCA1-2 *point mutations, led to the identification of five out-of-frame large rearrangements. Additionally, for the first time, a novel large rearrangement in the *BRCA2 *gene (Δ3 LR) that leads to a complete *in frame *deletion of exon 3 has been identified. The pathogenicity of exon 3 deletion is questionable. Exon 3 with 249 bp is in phase, similar to exons 10, 11, 12, 19 and 26, and therefore, deletion does not alter the reading frame and the functionally important amino-terminal BRCA2 region. The functional role of the affected protein exon 3 domain is not yet fully established and more striking, a physiological alternative transcript that lacks exon 3 (Δ3 delta3-transcript) has been described [4]. We report also here all of the variants of exon 3 and surrounding introns that were identified in 2461 index cases screened for germ-line mutations in both *BRCA1 *and *BRCA2*. Analysis of the transcripts of some of these variants is informative about the potential pathogenicity of exon 3 deletion. Finally, we propose three technical approaches that would contribute to a better understanding of the impact of variants on exon 3 skipping.

## Methods

### Probands

Genetic testing was offered to high-risk individuals according to criteria designed by the French national Group "Genetique et Cancer" (GGC) and Inserm recommendations [5]. Blood samples were collected following informed consent for participation in testing and subsequent research studies. The study was performed in the diagnostic context. The informed consent form was written and approved by a local medical ethical committee in accordance with guidelines from the French national group "Genetique et Cancer". A consecutive series of 2461 index cases (probands) were analyzed from 1996 to 2009 in our two French Medical Centres (Centre Paul Strauss, Strasbourg and Institut Curie-Hospital René Huguenin, St Cloud-Paris). We report here all of the variants identified in exon 3 of the *BRCA2 *gene. Large rearrangements in the *BRCA2 *gene were studied retrospectively in 2058 consecutive index cases that were found to be non informative (without any deleterious variant) after conventional *BRCA1/BRCA2 *mutation screening and *BRCA1 *large rearrangement analysis by MLPA.

Semi-quantitative experiments were performed on RNA derived from blood samples or lymphoblastoid cell lines (LBC) of index cases with mutations in *BRCA2 *exon 3 and surrounding introns. These included: in intron 2, c.68-7T>A variant (four proband samples), c.68-7delT (one proband sample) and c.68-7_8delinsAA (one proband sample); in exon 3, five patient samples carrying respectively the nonsense mutations c.71T>A (p.Leu24X), c.244A>T (p.Lys82X) and c.250C>T (p.Gln84X) and the missense variants c.125A>G (p.Tyr42Cys) and c.223G>C (p.Ala75Pro); in intron 3, one proband carrier of the c.316+3delA mutation. Eighteen control RNA samples, consisting of "PAXgene blood" (8 samples) and lymphoblastoid cell RNA (10 samples) from individuals screened non informative (without any deleterious variants) for *BRCA1/BRCA2 *genes were used.

To evaluate the proportion of delta3-transcripts in breast tumour tissues, cDNA samples from 185 sporadic invasive ductal breast carcinomas were analyzed. cDNA from three head and neck (HN) cancer tissues and three matched normal HN tissues were also analyzed to determine if this alternative transcript could be unbalanced in other tumour tissue types.

### BRCA2 large rearrangement screening

Large rearrangements (LRs) of the *BRCA2 *gene were investigated with two techniques: QMPSF assay (Quantitative Multiplex PCR of Short Fluorescent Fragments) using the conditions described by Tournier et al. [6,7] or MLPA (Multiplex Ligation-Dependent Probe Amplification) using the SALSA P045 BRCA2 MLPA kit (MRC Holland, the Netherlands) [8]. Results were evaluated by cumulative comparison of the samples with three normal control samples, after normalization relative to the peaks of the control gene and the control samples. All abnormal observation was confirmed with a second "alternative" technique and on a second independent blood sample from the patient.

### Characterization of the exon 3 large rearrangement

#### Zoom-in dedicated CGH array

To confirm and characterize large rearrangements of the *BRCA2 *gene, a zoom-in dedicated CGH-array was applied. An 11 kb-oligonucleotide microarray was specially designed using oligonucleotides designed in house and with validated oligonucleotides from Agilent (Agilent technology, USA), as described [9]. Of these, 9294 were located throughout the genome (4339 Agilent oligonucleotides and 3107 *BRCA1 *in-house oligonucleotides). The remaining 2749 oligonucleotides were specifically designed for the *BRCA2 *gene and its flanking regions. The region covered by the oligonucleotides was from 31,718,050 to 31,950,008 (Hg18, Human genome assembly 18). In the 5' region, there were 956 oligonucleotides that cover 70 kb. In the coding region, there were 1167 oligonucleotides that cover 84 kb. In the 3' region, there were 626 oligonucleotides that cover 78 kb. The analytical approach has been described elsewhere [9]. For the interpretation of the oligonucleotide signal, the threshold chosen was deleted if the log2 ratio was < -0.4 and duplicated if > 0.4. The log2 ratio was normalized as described in [9].

#### DNA breakpoints analysis

The breakpoints were characterized by long-range PCR of genomic DNA using the Qiagen Long-Range PCR kit (Qiagen, Germany). 150 ng of DNA was amplified with a primer pair that tightly flanks the respective breakpoint regions identified by the CGH-array in a reaction volume of 25 μl (Table 1). PCR products were subsequently fractionated through 1% agarose gels. The bands were purified with the QIAquick gel extraction kit (Qiagen, Germany), and sequenced with the same primers using the Big Dye terminator cycle sequencing reaction kit and an ABI-3130XL genetic analyser (Applied Biosystems, France).

**Table 1:** Oligonucleotide primers used for the exon 3 large rearrangement and transcript analysis

*BRCA2 *Primers	Nucleotide sequence	Size of PCR products	PCR temperature
	*Breakpoint analysis*		
C2del3-F2	5'_CAAGATCACTTCATTGATTTGTGAG_3'	4572 bp	59°C
C2del3-R2	5'_CGCTATATTTCTGTGTGCCTTTAAT_3'		
			
	*Transcript analysis*		
*Fragment analysis*			
Exon 02-06		480 bp	56°C
Rc2-01-F2	5 '6FAM_GATCCAAAGAGAGGCCAAC_3'		
Rc2-06-R	5'_CAAACTCCCACATACCACTGA_3'		
Exon 02-10		1350 bp	56°C
Rc2-01-F2	5' 6FAM _GATCCAAAGAGAGGCCAAC_3'		
Rc2-10R	5'_TGGTAGGCTAGAAATACGTGGC_3'		
*Pyrosequencing*			
*Allelic expression of c.-26G > A*			
Ex1-2 F	TACTCCGGCCAAAAAAGAACTGCA		
Ex2-Ex2/4 R	TATGTCTACTATTGGGAACATTCCTTCCTG	166 pb	55°C
Ex2-Ex3/4 R	ATTGGGAACATTCCTTCCTAAGTCTA	411 pb	55°C
Ex2 R	GATCCAATAGGCATTTTTACCTACGATATTCC	89 pb	55°C
Ex2 SEQ R	TACCTACGATATTCCTCCAATG		
*Competitive quantitative PCR*			
EX2 Cpcr F	TGAAATTTTTAAGACACGCTGCAACA		60°C
EX2/3 Cpcr R	AAACCAATTAAGACTTATTGGTCCTAAATCT	59 bp	60°C
EX2/4 Cpcr R	ACTATTGGGAACATTCCTTCCTGCT	47 bp	60°C
EX2 Cpcr SEQ F	TTAAGACACGCTGCAA		

#### Transcript Analysis

Depending on the availability of the material, total RNA was extracted either from blood samples collected in PAXgene Blood RNA tubes, using the PAXgene kit according to the manufacturer's protocols (Preanalitix, Qiagen, Germany) or from lymphoblastoid cell lines (LBC) using the acid-phenol guanidinium method with RNA-BTM solution (Eurobio, France).

An ubiquitous alternative transcript lacking exon 3 has already been described [4] but subsequent data on expression of this alternative transcript was discrepant [10-12]. In order to determine the effect of the Δ3 *BRCA2 *rearrangement and assess its pathogenic effect, we used three methods of quantification.

1) Fragment analysis: exon specific RT-PCR was performed with the Qiagen Onestep PCR kit as recommended (Qiagen, France) with a set of FAM-labelled primers spanning exon 2 to 6 and exon 2 to exon 10 (Table 1) for 25 cycles and including 200 ng RNA in 25 μl reaction volume. The products were analyzed with fragment analysis software after separation on a ABI-3130XL genetic analyser (Applied Biosystems, France). The experiments were performed in triplicate. This technique detects low levels of products and gives a semi-quantitative evaluation of the PCR products. Peak heights corresponding to exon exclusion and wild type transcript were measured and the proportion of the delta3-transcript was expressed as a percentage of the total amount of transcripts.

2) Allelic expression: allelic specific expression was evaluated by pyrosequencing with a PyroMark Q96 ID (Qiagen, France) with cDNA samples isolated from individuals who are heterozygous for the 5'UTR variant c.-26 G>A in exon 2 of the *BRCA2 *gene. This polymorphism is well documented and is called rs1799943 (37% of heterozygosity). Three sets of primers were used (Table 1): exon 2 to the junction between exons 3 and 4 (Ex2-Ex3/4) for the full-length transcript, exon 2 to the junction between exons 2 and 4 (Ex2-Ex2/4) for the delta3-transcript, and only on exon 2 for all transcripts. The proportion (%) of each allele was estimated for samples that are heterozygous for the c.-26 G>A variant by pyrosequencing using the EX2-SEQ primer. The heterozygosity was validated with the complete set of primers for all the transcripts. The robustness of this approach was validated with 10 control wild type samples; equivalent expression of each allele should result in a 50%/50% ratio, whatever the primer set used. For missense or nonsense mutations in the exon, the allelic expression of the specific heterozygous variant was also studied when the sample was not heterozygous for the c.-26 G>A (primer sequences on demand).

3) Competitive quantitative RT-PCR (Additional file 1): a competitive quantitative RT-PCR (C-QPCR) was set to detect imbalances of expression between the delta3 and full-length transcripts. The common forward primer (EX2 Cpcr F) was complementary to exon 2, the reverse primers were complementary to the exon2-exon3 junction (EX2/3 Cpcr R for the full-length transcript) and the exon2-exon4 junction (EX2/4 Cpcr R for the delta3-transcript) (Table 1). The primer mix contained 0.16 μM EX2 Cpcr F, 0.4 μM EX2/3 Cpcr R and 0.16 μM EX2/4 Cpcr R. These concentrations compensated for differences in the efficiencies of the PCR. Serial dilutions of mixtures of the two templates (delta3 and full-length) were used to validate the quantitation (Additional file 1c). The concentration was assessed by pyrosequencing with a PyroMark Q96 ID (Qiagen, France). The sequencing primer, EX2 Cpcr SEQ, is close to the exon2-exon3 junction, and the pyrosequencing sequence is CAGAT (Additional file 1a). The sequence of the full-length transcript is CAGAT and the delta3-transcript CAGGA without a T. The measurement of the proportion of delta3- and full-length transcript was done on the GA which is one for each nucleotide in the full-length transcript and two for each nucleotide in the delta3-transcript. The last T was a marker whose value is three in the full-length transcript and null in the delta3-transcript. cDNA samples from 96 lymphoblastoid cell lines from patients without mutations in exon 3 and intronic regions were used to validate the approach. The proportion of the delta3-transcript in the controls was 4% +/- 5%. All experiments were performed in triplicate.

Human Genetic Variation Society (HGVS) guidelines were followed for the nomenclature of *BRCA2*. The genomic positions correspond to the Human Genome 18/build 36 (2006). GenBank accession number used was NM_000059.3 and NP_000050.1 for cDNA and amino acid numbering respectively. The A of the ATG translation initiation codon is + 1.

## Results

### Point mutations in exon 3

Among the 2461 index cases analysed in the two Medical Centres, 35 variants were identified in exon 3. There were 5 nonsense mutations: c.71 T>A, (p.Leu24X), c.172G>T (p.Glu58X), c.244A>T (p.Lys82X), c.250C>T (p.Gln84X), c.273C > G (p.Tyr91X), and one deletion with a frameshift c.488_489del (p.Ser163IlefsX19). There were also several missense variants of unknown significance: nine c.125A > G (p.Tyr42Cys), one c.179A > G (p.Asn60Ser), three c.223G > C (p.Ala75Pro), one c.266C > T (p.Pro89Leu). In intron 2, there were nine c.68-7T > A, one c.68-7delT and one c.68-7_8delinsAA. In intron 3, there were 4 variants: c.316+3delA, c.316+5G > C, c.316+9T > A and c.316+33C > G. Unfortunately, RNA was only available for these variants: c.316+3delA, c.125A > G, c.223G > C, c.71T > A, c.244A > T, c.250C > T, c.68-7T > A, c.68-7delT, c.68-7_8delinsAA.

### Large rearrangement screening

Among the 2461 index patients, 2058 were analysed for large rearrangements in the *BRCA2 *gene by QMPSF and/or MLPA. Six large rearrangements were detected in the *BRCA2 *gene: one deletion of exon 3 (family with six breast cancers cases), two deletions of exons 15 and 16 (family with five breast cancer cases and in a breast/ovarian cancer family), one deletion of exons 14 to 18 (family with four breast cancer cases), one deletion of exons 22 to 27 (family with two breast cancer cases) and one duplication of exons 17 to 20 (breast cancer family). All, but the exon 3 deletion, led to frameshifts that alter the coding capacities of the alleles.

### Characterization of exon 3 deletion

**Figure 1:**
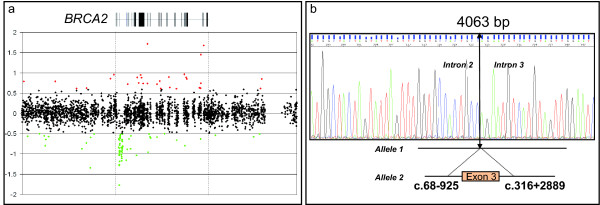
**Genomic analysis of the Δ3 *BRCA2 *large rearrangement**. a: *Dedicated BRCA2 CGH array*. The gene is represented at the top, with vertical boxes that indicate exon positions and sizes. Black plots are considered to be within the diploidy range (the y axis gives the log2 intensity ratios). The green dots indicate signals that were below the threshold for deletion (-0.4 log2 ratio). b: *Sequence analysis *of the smaller PCR product obtained by long-range PCR of proband DNA with the exon 3 large rearrangement in *BRCA2 *(Hg18/build36, 2006). The sequence crosses the breakpoint that begins in intron 2 and ends in intron 3.

Zoom-in dedicated CGH-array to the *BRCA2 *gene described the deleted region to measure from 3.6 to 6.7 kb (Figure 1a). The breakpoints and precise size of the deletion were established by long-range PCR. The primer set used gave rise to the expected normal PCR product of 4,572 bp in the control DNA. In the mutant DNA, a lower product of approximately 500 bp was observed in addition to the normal PCR product. Sequencing of the lower PCR product (Figure 1b) showed that the deletion spans 4,063 bp and includes 925 bp of intron 2, the entire exon 3 (249 bp) and 2,889 bp of intron 3. The rearrangement (Δ3-LR) is formally described as g.31.790.289_31.794.351del, and the deletion c.68-925_316+2889del, p.Asp23_Leu105del.

Two repeat elements around this deletion were reported. In the 5' region, there is a 735-bp sequence that is called Charlie 1a, type MER1, and in the 3' region a 313-bp sequence called L1MC2, LINE type. We did not detect any similarities in these two regions, either 1000-bp around the breakpoint or 1500-bp within the deletion.

In the proband RNA sample carrying exon 3 deletion, Onestep RT-PCR with primers spanning exons 2 to 6 gave two PCR products: the expected wild type product of around 480 bp and a lower sized product of ~ 200 bp. Both bands were of similar intensity. Sequencing of the smaller PCR product showed that it contains an *in frame *deletion of exon 3. Thus, the large genomic rearrangement Δ3-LR results in a stable delta3-transcript.

### Transcript analysis

In order to determine the effect of the Δ3-LR alteration and assess its pathogenic effect, semi-quantitative experiments were performed on various RNA samples. Three approaches were necessary to obtain unambiguous results and to determine the effects of sequence variants on delta3-transcript expression.

**Figure 2:**
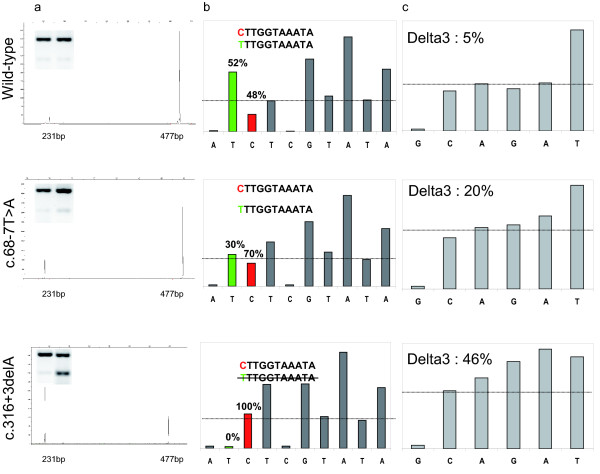
**Analysis of delta3-transcript expression**. a: *Fragment analysis of Onestep RT-PCR products spanning exon 2 to 6*. Semi-quantitative fragment analysis of Onestep RT-PCR products revealed the presence of the wild type transcripts at 477 bp and the delta3 alternative transcripts at 231 bp, as shown in the electrophoretograms of a control sample (wild type) and samples with the c.68-7T>A and c.316+3delA mutations. The percentages of delta3-transcript were, respectively: 6%, 22% and 61% for the sample illustrated. Left upper panels: agarose gel images of the same products after 33 cycles of PCR; the left wells contain the control, the right wells the samples with the respective mutation. b: *Pyrosequencing histogram from the pyrogram for the c.-26A>G polymorphism *with the primer set Ex1-2 F and Ex2-Ex3/4 R (only the full-length transcript). The sequence is C/T TTGGTAAATA. The proportion of C and T is directly computed by the pyrosequencing software. c: *Pyrosequencing histogram *from the pyrogram *for the competitive QPCR *using primer set exon 2/exon 3 and exon 2/exon 4 for the full-length transcript and delta3-transcripts, respectively. The sequence of the exon 2/3 transcript is CAGATTT and the sequence of the exon 2/4 transcript is CAGGAA. The proportion of GA (full-length transcript) and GGA (delta3-transcript) is directly computed on the pyrosequencing software.

Semi-quantitative fragment analysis of Onestep PCR products spanning exons 2 to 6 was necessary, since products fractionated on agarose gels do not give reliable results. In particular, the abnormal shorter PCR product, corresponding to the delta3-transcript, was very faint and not always detectable (Figure 2a). This observation could explain why Machado et al. [11] and Peixoto et al. [12] report discordant results. Fragment analysis with a fluorophore on an automatic sequencer gave reliable results, which were confirmed with PCR products spanning exons 2 to 10 (data not shown). As shown in Table 2 and Figure 2a, the proportion of the delta3-transcript to the total transcript was variable according to the type of variant. The "control samples" (i.e. wild type or not mutated in exon 3), gave low levels of the delta3-transcript that was, on average, less than 10% of the total transcript. In contrast, with samples from cases carrying the Δ3-LR or the c.316+3delA, the PCR products corresponding to the delta3-transcript corresponded to 61% and 52% of the total. Samples with nucleotide variants in intron 2 also had increased levels of the delta3-transcript, which proportions ranging from 14% to 52% of the total. Heterogeneity within samples carrying the same variant was also observed. The discrepancy for c.68-7T>A could be associated with the nature and quality of the cDNA from lymphoblastoid cells (52% and 45%) or from PaxGene extraction (14% and 23%). Samples with nonsense mutations in exon 3 gave a level that was similar to control samples, with a range between 5% and 12%. The results were similar when samples were treated with puromycin. Thus, two classes of expression were detected, with low or high levels of delta3-transcripts. In this approach, competition in the PCR between the two amplicons with different sizes could result in preferential amplification of the lower sized delta3-transcript. To circumvent this problem, we developed two alternative methods: allele-specific RT-PCR and competitive quantitative RT-PCR (C-QPCR) combined with pyrosequencing.

**Table 2:** Analysis of delta3-transcript expression by fragment analysis, allele specific PCR on c.26 G>A heterozygous samples and competitive quantitative C-QPCR

	**Fragment analysis** % delta3-transcript /total transcripts (± standard deviation)	**Allelic discrimination** Exclusively on exon3 full-length transcript (± standard deviation)		**Competitive QPCR** % delta3-transcript /full-length transcript (± standard deviation)
		**A allele**	**B allele**	
Wild-type (10 samples)	8 ± 2% *	48 ± 4%A	52%G	4 ± 2%
c.316+3delA	52 ± 7%#	0% A	100%G**	44 ± 4%
Delta3 BRCA2 rearrangement	61 ± 3%#	-	-	44 ± 7%
*Intron 2 variants*				
c.68-7T>A	14 ± 2%#	-	-	31 ± 10%
c.68-7T>A	23 ± 4%#	-	-	25 ± 4%
c.68-7T>A	52 ± 2%	27 ± 6%A	**73%G**	23 ± 3%
c.68-7T>A	45 ± 12%	33 ± 3%A	**67%G**	27 ± 3%
c.68-7delT	22 ± 2%#	28 ± 2%A	**72%G**	32 ± 5%
c.68-7_8delinsAA	49 ± 1%	**72 **± 10**%A**	28%G	25 ± 2%
*Missense mutations*				
c.125>G, p.Tyr42Cys	6 ± 1%	47 ± 7%A**	53%G**	3 ± 1%
c.223G>C, p.Ala75Pro	3 ± 1%	50 ± 1%G**	50%C**	5 ± 2%
*Nonsense mutations*				
c.71T>A, p.Leu24X	5 ± 1%	52 ± 2%T**	48%A**	2 ± 1%
c.244A>T, p.Lys82X	11 ± 1%	52 ± 4%A**	48%T**	11 ± 2%
c.250C>T, p.Gln84X	12 ± 1%	57 ± 4%C**	43%T**	10 ± 6%

# RNA from PAXgene, *three RNA samples from PAXgene and seven RNA samples from lymphoblastoid cells, ** heterozygosity was proven with other set of primer. Values (%) are means of three experiments.

Some of the samples that are heterozygote for the c.-26 G>A polymorphism of the *BRCA2 *gene were analyzed by pyrosequencing, to determine the proportion of transcripts that come from each allele (allelic discrimination) and quantify the relative proportion of delta3-transcript (Table 2 Figure 2b and Additional file 2). For wild-type samples (c.-26 G>A heterozygous), as expected, the percentage of G and A alleles was close to 50%, whatever the primer set used. For example, with the primer that spans the exon 3-4 junction, the average was 48% A and 52% G, with a standard deviation of 4%. Significantly, for the c.316+3delA, one allele expressed the full-length transcript and the other the delta3-transcript, suggesting exclusive expression of the delta3-transcript from the mutant allele. Unfortunately, there were no c.-26 G>A polymorphism in carriers of the Δ3-LR. By definition, the allele with the Δ3-LR cannot express the full-length transcript. This sample was then used as a reference. For variants in intron 2, the percentages of the A (or G) and G (or A) allele were 30% and 70%, respectively, using sets of primers specific to the delta3- and full-length transcript, whereas they were 50%-50% with the set of primers for all transcripts, suggesting that there is an imbalance in favour of the delta3-transcript. The allelic discrimination was quantified also with variants other than c.-26 G>A, including c.71T>A (p.Leu24X), c.125A>G (p.Tyr42Cys), c.223G > C (p.Ala75Pro), c.244A > T (p.Lys82X), c.250C > T (p.Gln84X) within exon 3. For the missense variants [c.125A > G (p.Tyr42Cys) and c.223G > C (p.Ala75Pro)], the quantification gave a balanced proportion of A and G (47-53%) and G and C (50-50%) respectively (Table 2). For the nonsense variants [c.71T>A (p.Leu24X), c.244A>T, (p.Lys82X) and c.250C>T (p.Gln84X)], the results were comparable to wild-type, (50% for each allele). Puromycin treatment did not change the results, suggesting that nonsense mediated mRNA decay (NMD) is not involved.

Competitive quantitative RT-PCR (C-QPCR) analysis confirmed the results from fragment analysis. Due to the higher sensitivity of this method, the results appear to be more reliable. The values were globally lower than by fragment analysis, and could differentiate between three classes of delta3-transcript levels (Table 2, Figure 2c and Additional file 1). For wild-type samples, the proportion of delta3-transcript was close to 4%. As in fragment analysis, the ratio between delta3- and full-length transcripts was significantly higher, close to 40%, for both mutations leading to exclusive deletion of exon 3. For intronic variants in intron 2, the proportion was intermediate, close to 25%. There is less disparity within samples, compared to fragment analysis. For the missense variants, c.125A>G (p.Tyr42Cys) and c.223G>C (p.Ala75Pro), the proportion was similar to wild type, 3% and 5%, respectively. For the nonsense variants, the proportion ranged from 2 to 10%, consequently they can be included to the class with low levels of delta3-transcript expression.

On 185 sporadic breast cancer samples, C-QPCR gave low (< 10%) to no detectable expression of the delta3-transcript, except for seven samples (4% of samples analysed). The increases of delta3-transcripts were 10-15% in four samples, 15-20% in 2 samples and about 45% in one. This latter sample, with significant expression of delta3-transcript, came from an inflammatory triple negative breast cancer (0.5% in the samples analysed). Constitutional analysis could not be performed, to determine the *BRCA1/2 *status of the patient.

### Co-segregation analysis

To determine whether the exon 3 deletion co-segregates with cancer, we examined the occurrence of this allele in members of the family A-2005 with the Δ3-LR and of the family B-2002 with the c.316+3delA mutation.

**Figure 3:**
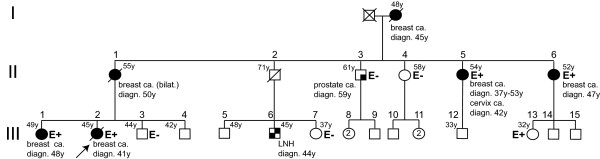
**Family pedigree (A-2005) of the patient carrying the *BRCA2 *Δ3 large rearrangement**. Pedigree symbols: *black figures: *affected individuals, *diagonal slash: *deceased individuals; *arrow: *proband, E: tested for Δ3 large rearrangement, E+: mutation carrier, E-: non carrier, LNH: Non-Hodgkin lymphoma, ca.: cancer.

Family A-2005 (Figure 3) had a breast cancer family history with 6 assessed breast cancer cases. Four of them, of which the proband (III-2), could be tested and were carriers. The affected mother of the proband could be considered as an obligate carrier. Added to a non affected female cousin (32 years old) who tested positive, the carrier genotype could be established or deduced in six females. Out of them, 5 had developed breast cancer at age 37, 41, 47, 48 and 50 (mean age: 44.6), with bilateral breast cancer in one case (II-5). One female carrier underwent bilateral prophylactic mastectomy at age 32. Penetrance could be estimated at 50% by age 45 and at 100% by age 50. Histopathological data, when available, showed grade II or III, oestrogen-receptor (ER) positive and HER2-negative breast tumours, a phenotype consistent with *BRCA2*-associated breast cancers. Thus, in this family, the Δ3-LR allele segregated well with affected family members, which is consistent with the hypothesis that Δ3-LR is a deleterious allele.

**Figure 4:**
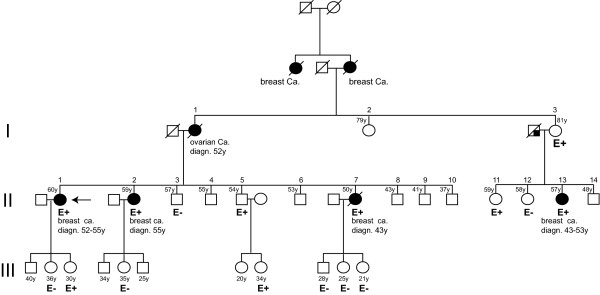
**Family pedigree (B-2002) of the patient carrying the *BRCA2 *c.316+3delA mutation**. Pedigree symbols: *black figures: *affected individuals; *diagonal slash: *deceased individuals; *arrow: *proband, E: tested for c.316+3delA mutation, E+: mutation carrier, E-: non carrier, ca.: cancer

Family B-2002 (Figure 4) is a breast/ovarian cancer family, with four breast cancer cases at 43, 43, 52 and 55 years old (mean age: 48.3) and one ovarian cancer at age 52. Controlateral breast cancer occurred in two patients (II-1 and II-13). The c.316+3delA mutation of *BRCA2 *was detected in the proband (II-1) affected with breast cancer at age 52, controlateral breast cancer at 55, and also papillary thyroid cancer at age 33. The mother with ovarian cancer at 52 is an obligate carrier. Histopathological features, when available, consisted of high grade breast tumours, ER and PR positive, HER2 negative. Familial analysis revealed four unaffected female carriers 30, 34, 59 and 81 years old. Except for one unaffected carrier, who underwent bilateral prophylactic annexectomy at age 57 (II-11), no risk reducing surgery was performed among the three other carriers. Penetrance could be estimated at 30% by age 45 and 70% by age 60.

As a whole, segregation analysis indicates a high breast cancer risk (100% and 70% by age 60) in both families, and argues in favour with a deleterious effect of exon 3 skipping in mutation carriers.

The segregation data for the nonsense mutations in exon 3 that we studied were too limited to allow conclusions about penetrance and comparison with the afore-mentioned mutations. In brief, the individual with p.Gln84X (c.250C>T) had an ovarian cancer at 56 years-old and a breast cancer at 59 years-old. Her mother had a fallopian tube cancer at 62 years-old and a breast cancer at 66 and her grand-mother a breast cancer around 62. The individual with p.Lys82X (c.244A>T) had a breast cancer at 44 years-old and her mother, who also carries the mutation, a breast cancer at 45 and an ovarian cancer at 65. Her mother's sister had also a breast cancer at 45. The individual with p.Leu24X (c.71T>A) had a breast cancer at 45 years-old and belonged to a family with several breast cancers in the maternal branch (mother: 74, aunt: 50, aunt: 40). Her sister is a carrier and had a bladder cancer at 39. Finally, all carriers were affected by breast cancer between 44 and 66 years-old.

## Discussion

The Δ3-LR described here has not been reported previously and is the third reported mutation that leads to total exon 3 deletion of the *BRCA2 *gene. The c.277.317-726delinsCCAT (putative p.Ser98Glu106delinsPro) has been detected in a Swedish breast/ovarian cancer family [13]. The c.156_157insAlu has been described in a Portuguese family as an Alu insertion at codon 52 of *BRCA2 *[14,15] and is now considered to be a frequent founder mutation that is detected in nearly one third of breast/ovarian cancer families from northern/central Portugal [16,17]. In our large series of 2058 cases that are non informative for *BRCA1-2 *point mutation, the prevalence of *BRCA2 *large rearrangements (LR) is estimated to be 3% of all *BRCA2 *deleterious mutations. This value is lower than the 7% to 11% reported previously [3,7,18]. In our six pedigrees, there were no male breast cancer cases, which contrasts with the literature [7]. Moreover, the low frequency of *BRCA2 *LR could be explicable by the lower frequency of Alu sequences in the genomic locus of *BRCA2 *in comparison to *BRCA1*. Screening for *BRCA2 *large constitutive rearrangements should be recommended in comprehensive genetic tests, especially for families with multiple breast and/or ovarian cancer cases and families with at least one case of male breast cancer.

There is some debate about the pathogenic effect of *BRCA2 *exon 3 alterations due to the particular features of exon 3 in *BRCA2. E*xon 3 is in phase in the gene and is absent in the physiological alternative transcript (delta3-transcript) that has been detected in normal tissues, and in particular at moderate to low levels in mammary gland and prostate tissues [4].

We extensively analysed the balance between delta3- and full-length transcript levels, using different methods that include allele specific expression and competitive quantitative PCR by pyrosequencing which avoids misinterpretations associated with fragment analysis. We observed three classes of differential expression of the delta3-transcript.

We detected low levels of the delta3-transcript in wild-type RNA, which represent less than 10% of the total transcript levels, which could be considered to be constitutive expression of the delta3-transcript. Alternative splicing is less common for *BRCA2 *than for *BRCA1*. A splice variant lacking exon 12 of *BRCA2 *has been detected at higher levels in 33% of breast tumours compared to matched normal tissues, suggesting that dysregulated expression of the isoform may contribute to breast cancer progression [19]. However, recent data indicate that exon 12 is redundant and its loss may not have an impact on the disease [20]. We detected low levels (nearly 10%) of the alternative transcript that lacks exon 3 *BRCA2*, using 185 sporadic breast tumours samples and some head and neck tumours and normal tissues samples. This constitutive low level of expression suggests that the delta3-transcript is not involved in tumorigenesis in sporadic tumours. The physiological role of this low level of the delta3-transcript remains to be established.

In the case of the Δ3-LR, genomic deletion of exon 3 results in an increase of the proportion of the delta3-transcript relative to the full-length transcript, to 40-50% (by fragment analysis and confirmed by competitive QPCR). This corresponds to a total loss of exon 3 on one allele, as expected due to the large rearrangement. The same result was observed for the sample with the c.316+3delA mutation that also leads to the loss of exon 3 in the RNA. Moreover, the exclusive expression of delta3-transcript by the mutated allele, which we demonstrated by allelic discrimination analysis of the c.316+3delA mutation that is heterozygous for the c.-26 G>A polymorphism, confirms our results. Our results are similar to those reported for a mutation in intron 3, c.316+5 G>C (which was analyzed in a mini-gene system [21]) and the Portuguese Alu insertion [16], both of which result in total splicing out of exon 3 on the mutated allele. Consequently, these data, taken together, support the hypothesis of a pathogenic effect when there is exclusive synthesis of the delta3-transcript from one allele. Such functional mRNA splice variants have been demonstrated for two splice site mutations in *BRCA1 *gene, c.212+3A>G and c.135-6T>G. These isoforms, that lead to an *in frame *deletion of exon 5, were found to be expressed at increased levels in tumour cells [22].

The hypothesis of a causal effect of exon 3 deletion is supported by the co-segregation analysis of both families, with Δ3-LR and c.316+3delA, respectively. Co-segregation with disease of loss of exon 3, or of total exclusion of the full-length transcript, is strong, and results in high penetrance of breast cancer in mutation carriers of both families. This is also the case for the afore mentioned mutations, c.316+5G>C [21] found in all affected members of two families with breast and breast/ovarian cancer syndrome (S. Krieger personal communication) and the founder Portuguese mutation c.156_157insAlu [14,16,17]. Taken together, these data highlight the clinically relevant effects of the Δ 3 mutant allele and indicate that it is associated with a significant breast cancer risk.

Our analysis of some intron 2 variants also supports our contention that loss of exon 3 is relevant for breast cancer risk. These variants (c.68-7T>A, c.68-7delT, c.68-7_8delinsAA) have increased levels of the delta3-transcripts, which account for 23 to 30% of the total *BRCA2 *transcripts. There is an allelic imbalance of 30/70 between full-length transcript and delta3-transcript, that was shown with the heterozygous c.-26A>G samples. This proportion suggest that both alleles synthesise the two transcripts (full-length and delta3), with a slight imbalance for the mutated allele. However, previous reports as well as *in silico *predictions do not favour a causal effect of the intron 2 mutations. The c.68-7T>A variant, initially described in one patient with breast and ovarian cancer in Italy [23], has been widely detected in France (82 times, UMD-BRCA2 database [24]) sometimes with co-occurrence of two different deleterious *BRCA2 *mutations. The c.68-7delT has been previously described in four endometrial carcinomas with microsatellite instability, one of which had another pathogenic mutation in *BRCA2 *[25], and in three colorectal cancers with microsatellite instability [23]. It was detected once in our cohort, 54 times in France (UMD-BRCA2 database) [24] with co-occurrence of deleterious *BRCA2 *mutations, and 4 times in the BIC database [26]. Consequently, the slight increase in the delta3-transcript could be considered to be neutral in terms of high risk of breast/ovarian cancer. The relevance of intermediate levels of delta3-transcription for cancer risk remains to be established. In fact, in these cases, the loss of the wild-type allele in tumour tissue would lead to an allele which produces two forms of protein with and without exon 3 domain. Thus, we suggest these three distinct intron 2 mutations should remain unclassified variants until co-segregation analysis, evaluation of frequency in control population and/or functional studies are performed, which would clarify whether there is any risk associated with these mutations.

The results we obtained for the three nonsense mutations in exon 3 that we studied, and that we confirmed by the three transcript analysis methods, have not been described previously. No significant increase in the delta3/wild type transcript ratio was detected. This contrasts with the Portuguese Alu insertion mutation, which creates a stop codon 10 nucleotides after the insertion, but exclusively expresses the delta3-transcript. In fact, our results suggest that the mutated mRNAs, that contain the premature stop codon, are not eliminated or destabilized by NMD [27,28]. Indeed, if NMD were involved, the expression levels of full-length transcript from the mutant allele would have been significantly decreased, whereas the expression levels of the delta3-trancript issued from both alleles would probably not be modified, thus leading to a global increase of the proportion of the delta3-transcript.

An additional argument towards a pathogenic effect of Δ3-LR would reside in the impact of the loss of exon 3, at the functional level, especially in tumours that exclusively express the transcript due to the genomic deletion on one allele and loss of the wild type allele. The delta3-transcript codes for a putative protein that lacks an 83 amino acids highly conserved bipartite domain, including a primary activating region (PAR, amino acids 23-60) and an auxiliary activating region (AAR, 60-105) [29,30] whose transcriptional activity could be regulated by phosphorylation through two potential phosphorylation sites [31,32].

Of most relevance, PAR has been shown to interact with two proteins, EMSY and PALB2 (Partner and Localizer of BRCA2), which are involved in breast carcinogenesis. EMSY has endogenous transcriptional repressor activity and contributes to DNA damage focus formation [33]. Similar to BRCA2, EMSY relocates to double-strand break repair sites following DNA damage [34] and forms a complex with RAD51. EMSY can silence the transcription activation potential of the BRCA2 protein region encoded by exon 3 and thereby negatively regulate BRCA2 [33]. Our study shows that delta3-transcript expression is not significantly increased in sporadic breast tumours, suggesting that other mechanisms are involved in non-hereditary breast tumorigenesis. To date, the EMSY gene has been found to be amplified in 7-13% of sporadic breast cancers, 17% of ovarian cancers [35] and 13% of pancreatic cancers [36]. Excess of EMSY protein, that results from gene amplification, could contribute to the tumorigenesis of a substantial proportion of non-inherited sporadic breast and ovarian cancers, by silencing BRCA2 [37].

PALB2 is an integral component of the BRCA complex. It co-localises with BRCA2 in nuclear foci and promotes its stability in nuclear structures [38], which is essential for key BRCA2 nuclear caretaker functions. PALB2 also mediates the physical interaction of BRCA2 with a carboxy-terminal fragment of BRCA1 and is required for the cooperation between BRCA1 and BRCA2 in the DNA damage responses [39]. Loss of the exon 3 region could affect the intranuclear stability of a subpopulation of BRCA2 proteins that is required for DNA repair. This hypothesis is reinforced by the demonstration that "clinically unclassified" missense variants, located in exon 3 of *BRCA2 *(p.Trp31Arg, p.Trp31Cys, p.Gly25Arg), display complete loss or reduction of PALB2 binding activity and inefficient DNA repair [38]. In contrast, the p.Tyr42Cys (Y42C) variant did not differ from wild-type [38]. The p.Tyr42Cys variant has now been excluded as a disease associated *BRCA2 *variant, from in vitro functional studies [40], and genetic and epidemiologic data, i.e. absence of co- segregation [41] and high frequency [42 times in France (UMD-BRCA2)[24] and 141 times in BIC]. The low expression of delta3-transcripts we observed for this variant is in accordance with these conclusions. Taken together, our data lead to the hypothesis that disruption of the balance between *BRCA2 *transcripts could predispose to disease. BRCA2 protein, without the exon 3 region, may act as a dominant negative inhibitor of its transcriptional function, and could also be modified in its DNA repair activity.

## Conclusion

There are strong arguments to suggest that loss of the exon 3 encoded "region" could be a cause of breast cancer predisposition. The exon 3 region is involved in the transcriptional and DNA damage response functions of BRCA2. Furthermore exclusive expression of the delta3-transcript co-segregates with the disease. Our study suggests that exclusive transcript expression from the mutated allele contributes to high risk breast cancer, whereas moderate to low delta3-transcript levels are neutral as long as the full-length transcript is present at a roughly normal level. However, the question remains open whether low to moderate levels of the delta3-transcript have an impact on BRCA2 functions and could contribute to increased risk of breast cancer. Additional analysis of the functional consequences of loss of exon 3 in the *BRCA2 *gene are required, particularly for those variants that lead to intermediate delta3-transcript level. Noteworthy, although the transcript is deleted for exon 3, the highly conserved and functionally important amino-terminal *BRCA2 *region is preserved. Moreover, when investigating potentially lacking exon 3 mutations, various analysis approaches should be combined to differentiate their impact on transcript imbalance and co-segregation studies should be performed systematically to further support these data.

## Competing interests

The authors declare that they have no competing interests.

## Authors' contributions

DM and ER designed the study, interpreted the data and wrote the manuscript. IS and CL carried out the molecular analyses. SC analysed the UMD database. OC, CN, JML, LM and JPF provided families. JPF provided segregation data. JPF, RL and JA have revised the manuscript and have given approval for final submission. All authors read and approved the manuscript.

## Pre-publication history

The pre-publication history for this paper can be accessed here:

http://www.biomedcentral.com/1471-2350/12/121/prepub

## Supplementary Material

Additional file 1 **Schematic figure to explain the principle of competitive quantitative PCR (C-QPCR)**. a) pyrograph (right) and subsequent pyrogram (left) obtained using the two set of primers on exon 2 and exon 3 (full-length transcript) and on exon 2 and exon 4 (delta3-transcript). b) pyrograms of the C-QPCR showing the three levels of delta3-transcript (5%, 20%, 46%). c) sensitivity study of the C-QPCR obtained with a mix of exon2-3 amplicons and exon 2-4 amplicons for a range of delta3-transcript concentrations (5%, 10%, 25%, 50% 75%, 90%). On the × axis, the proportion of delta3-transcripts (exon2-4) in the mix, y axis, pyrosequencing results in % of delta3-transcripts from the C-QPCR. Click here for file

Additional file 2 **Illustration of the consequences on proportion of delta3-transcripts and on allelic discrimination, of the three cases: wild-type, moderate (c**.68-7T>A) and exclusive (c.316+3delA and exon 3 deletion) expression of exon 3 spliced transcripts. The A/B allelic discrimination panel gives the theoretical percentages of the allele depending on the position of primers that specifically dose the total, the delta3- or the full-length transcripts. Click here for file
